# A novel method to purify small RNAs from human tissues for methylation analysis by LC-MS/MS

**DOI:** 10.3389/fmolb.2022.949181

**Published:** 2022-08-30

**Authors:** Rong Yang, Jianfeng Li, Yifan Wu, Xinli Jiang, Shuang Qu, Qiang Wang, Hongwei Liang, Ke Zen

**Affiliations:** ^1^ State Key Laboratory of Pharmaceutical Biotechnology, School of Life Sciences, Nanjing University, Nanjing, China; ^2^ State Key Laboratory of Pharmaceutical Biotechnology, School of Life Sciences, Nanjing University, Nanjing, China; ^3^ School of Life Science and Technology, China Pharmaceutical University, Nanjing, China

**Keywords:** small RNAs, 2′-O-methylation, LC-MS/MS, lung tissue, sperm

## Abstract

Methylation modification of small RNAs, including miRNA, piRNA, and tsRNA, is critical for small RNA biogenesis and biological function. Methylation of individual small RNA can be defined by liquid chromatography-coupled with mass spectrometry (LC-MS/MS). However, LC-MS/MS analysis requires a high purity of individual small RNA. Due to the difficulty of purifying specific small RNA from tissues or cells, the progress in characterizing small RNA methylation by LC-MS/MS is limited. Here, we report a novel method that can efficiently purify small RNA from human tissues for LC-MS/MS analysis. This method includes two steps: 1) pull down the target small RNA by incubating total small RNAs (18–24 nt) extracted from human tissues with a biotinylated antisense oligonucleotide of the target small RNA, followed by capturing the binding duplex of biotinylated antisense and small RNA *via* streptavidin magnetic beads, and 2) protect the target small RNA by pairing it with a single-strand DNA, which sequence is complementary to the target small RNA, to form a DNA/RNA hybrid double-strand, followed by sequential digestion with exonuclease I, nuclease S1, and DNase I, respectively. Furthermore, employing a mixture of four pairs of synthetic methylated and non-methylated small RNAs, we further refined this two-step method by optimizing the nuclease S1 treatment condition. With this method, we successfully purified miR-21-5p, miR-26-5p, piR-020485, and tsRNA from human lung and sperm tissue samples and analyzed their 2′-O-methylation modification at the 3′-end by LC-MS/MS.

## Introduction

2′-O-Methylation (2′-Ome) or Nm (N stands for any nucleotide) is an essential post-transcriptional modification of RNA, in which a methyl group (-CH3) is added to the 2′-hydroxyl (-OH) of the ribose moiety (Dimitrova et al., 2019). The 2′Ome modification has been shown to affect RNAs in multiple ways, such as increasing RNA hydrophobicity (Kumar et al., 2014), protecting RNA against nuclease attacks (Ji and Chen, 2012), stabilizing helical structures (Yildirim et al., 2014), and affecting their interactions with proteins or other RNAs (Ji and Chen, 2012; Dimitrova et al., 2019). As an abundant and highly conserved modification in tRNA, rRNA, mRNA, and snRNA (Ayadi et al., 2019; Ontiveros et al., 2019), 2′Ome has been identified at the 3′-end of almost all types of cellular small RNAs, including miRNA and siRNAs in plants and miRNAs, piRNAs, and tsRNAs in animals and humans (Li et al., 2005; Yu et al., 2005; Ohara et al., 2007; Abe et al., 2014; Chen et al., 2016; Liang et al., 2020a).

Detection of 3′-terminal 2′Ome modification in individual small RNAs, however, has long been a challenge. LC-MS/MS and two-dimensional thin-layer chromatography (2D-TLC) are two platforms to determine the 3′-terminal 2′Ome in specific RNAs, such as tRNA and rRNA (Ayadi et al., 2019; Dimitrova et al., 2019). However, applying these techniques to analyze the 3′-terminal 2′Ome modification of small RNAs, like miRNA, is challenging. The main obstacle is to achieve the RNA purity required by LC-MS/MS. Although the direct RNA pulldown assay was employed by Yu et al. to isolate individual miRNA from *Arabidopsis* (Yu et al., 2005), the efficiency of direct small RNA pulldown assay is relatively low. Moreover, due to the great sequence similarity among different miRNAs, the purity of isolated individual miRNA *via* RNA pulldown assay is often not qualified for LC-MS/MS analysis. Currently, the identification of the 2′Ome of small RNAs mainly relies on sodium periodate oxidation followed by Northern blotting analysis (Yu and Chen, 2010; Ji and Chen, 2012). However, this method cannot identify the position of methylation in individual small RNA.

In the present study, we combined RNA pulldown assay and enzyme protection assay to develop a two-step strategy to isolate specific small RNA. Using this novel two-step method, we successfully purified specific small RNA including miRNA, piRNA, and tsRNA from tissue samples and accurately identified their 3′-terminal 2′Ome modification by LC-MS/MS.

## Materials and methods

### Tissue preparation

Human normal lung tissue and sperm samples were obtained from Nanjing Drum Tower Hospital of Nanjing University (Nanjing, China). This study has been approved by the Ethics Committee of the Nanjing Drum Tower Hospital of Nanjing University and a written consent form was obtained from each donor.

### Small RNA extraction, purification, and detection

As the previous reports (Guo et al., 2017; Sriram et al., 2021), the small RNA extraction kits did not perform as well as Trizol in the separation of miRNA. The total RNAs of the normal lung tissue and sperm samples were extracted with Trizol reagent (Invitrogen) according to the manufacturer’s instruction. To extract small RNAs of 18–24 nt, the large RNAs were removed from total RNAs by selective precipitation with 5% polyethylene glycol 8000 in 0.5 M NaCl. Small RNAs were then precipitated from the supernatants by adding three volumes of absolute ethanol and 0.1 volume of 3 M sodium acetate (pH5.2) (−80°C, overnight). The small RNAs were then separated by 15% PAGE containing 7 M urea (300V, 35 min). The gel was stained with SYBR Green I solution and visualized by gel documentation system (Wealtec). Small RNAs with a size of 18–24 nt were excised from the gel and then extracted using 0.3 M NH_4_Cl and precipitated with glycogen and ethanol. The quality of the purified small RNAs was determined on an Agilent 2100 Bio-analyzer using a small RNA Kit (Agilent).

### Synthetic small RNA oligonucleotides and primers

Four pairs of unmethylated and methylated (3′-terminal 2′Ome) human small RNAs (miR-21-5p/miR-21-5p^CH3^, miR-26-5p/miR-26-5p^CH3^, piR-020485/piR-020485^CH3^, and tsRNA/tsRNA^CH3^) were synthesized in GenScript (Nanjing, China). Oligonucleotides and primers were dissolved in DEPC water to generate 20 μM/μL and 100 nM/μL stock solutions and stored at −70°C, respectively. For stem-loop primer RT-qPCR calibration, the reaction concentration was 0.1–1.0 µM. The sequences of oligonucleotides and primers used in the experiment are listed in Supplemental Table S1.

### RT-qPCR

Hydrolysis probe-based qRT-qPCR assay for small RNAs was performed on an Applied Biosystems 7300 Sequence Detection System (Applied Biosystems) according to the manufacturer’s instructions. Briefly, the reverse transcription reaction was carried out in a 10 μL reaction buffer containing 1 µL of extract RNA, 1 µL of 10 mM dNTPs, 1 µL of AMV reverse transcriptase (TaKaRa), 1 µL of stem-loop RT probes/primers (Applied Biosystems), 2 µL of 5×reverse transcription buffer, and 4 µL of diethylpyrocarbonate (DEPC) water. For cDNA synthesis, the reaction mixtures were incubated at 16°C for 30 min, 42°C for 30 min, 85°C for 5 min, and then held at 4°C. Real-time PCR was performed (1 cycle of 95°C for 5 min and 40 cycles of 95°C for 15 s and 60°C for 1 min). The reaction was performed with a final volume of 20 µL containing 1 µL cDNA, 0.3 µL Taq, 0.33 µL hydrolysis probe (Applied Biosystems), 1.2 µL of 25 mM MgCl_2_, 0.4 µL of 10 mM dNTPs, 2 µL of 10 × PCR buffer, and 14.77 µL DEPC water. All reactions, including no-template controls, were performed in triplicate.

### Purification of small RNA

Purification of the target small RNA was carried out with a two-step strategy. First, total small RNAs were incubated with the biotinylated DNA oligonucleotide, which is antisense to the target small RNA such as miR-21-5p, miR-26-5p, piR-020485, or tsRNA in 0.5×SSC at 50°C for 15 h. Each biotinylated oligonucleotide (miR-21-5p probe: 5’−AAT​CAA​CAT​CAG​TCT​GAT​AAG​CTA−3′; miR-26-5p probe: 5′−AAAGCCT ATCCTGG ATTACTTGAA−3′; piR-020485 probe: 5′−GTC​CAG​GAG​ACA​CAC​TGT​CCT​GCA​CCA​AA−3′; tsRNA probe: 5′–GGG​GTC​GTA​AGC​CTC​TGT​TGT​CAG​ATT​CAC​AAT​C AA−3′) processed two extra A residues at its 5′-end to ensure that its molecular mass is larger than that of the target small RNA. The biotinylated DNA oligonucleotide was then captured by streptavidin magnetic beads (Roche) and washed with ×0.5 SSC. The target small RNA was then eluted with DEPC water by incubating at 70°C for 5 min. Second, a 2 µL single-strand DNA oligonucleotide (10 µM), which sequence is complementary to the target human small RNA (miR-21-5p: 5′-TCA​ACA​TCA​GTC​TGA​TAA​GCT​A-3′, miR-26-5p: 5′−AGCCTATCCTGGATT ACTTGAA−3′, piR-020485: 5′–GTC​CAG​GAG​ACA​CAC​TGT​CCT​GCA​CCA−3′ or tsRNA: 5′–GGGGTC GTA​AGC​CTC​TGT​TGT​CAG​ATT​CAC​AAT​C−3′), was mixed with the isolated target small RNA (10 µg) in buffer (20 nM Tris–HCl, 100 mM KCl, 5mM MgCl_2_, pH7.5), and then incubated at 95°C for 2 min, followed by slowly temperature-decreasing to 25°C. To eliminate the unpaired RNAs, 1000 units Nuclease S1 (Thermo Fisher) was added to the solution. The reaction was allowed for 30 min at room temperature and terminated by 70°C incubation. The ssDNA was then degraded by adding 4 units DNaseI (New England Biolabs) (37°C, 30 min). The target small RNAs (purified miR-21-5p, miR-26-5p, piR-020485 or tsRNA) were finally extracted by the RNA Clean and Concentrator-5 kit (Zymo Research) according to the manufacturer’s instructions. These nucleosides were then subjected to LC-MS/MS analysis using a 4600 UPLC/Triple TOF (Kullolli et al., 2014; Konno et al., 2019; Zhang et al., 2019; Liang et al., 2020b; Feng et al., 2022). Mass spectrometry conditions were set as previous report (Kullolli et al., 2014): ion transfer tube temperature: 150°C; spray voltage: −1.8 kV; activation time: 10 ms for CID and 30 ms for HCD.

## Results

### Two-step strategy to purify small RNA

**FIGURE 1:**
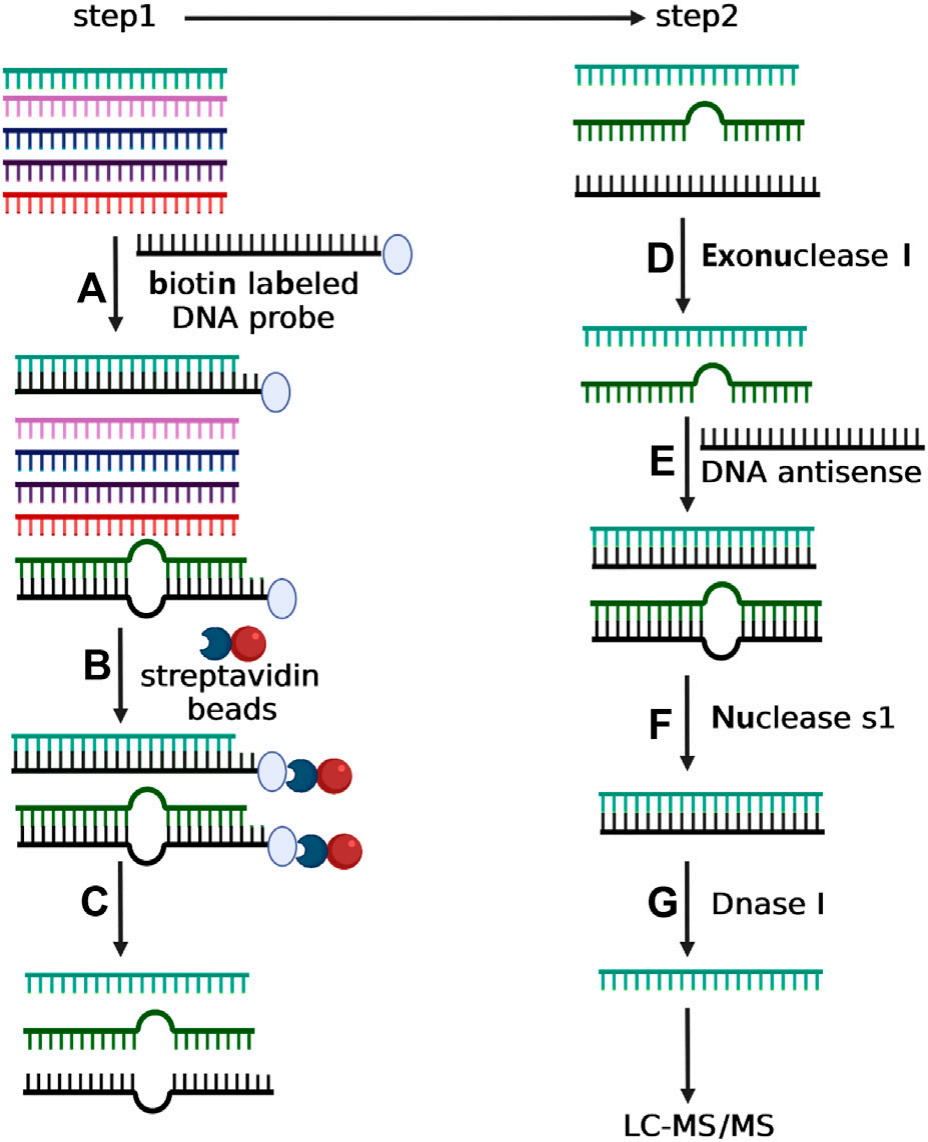
Depiction of two-step experimental strategy for isolating selective small RNA. In step 1, total small RNA was incubated with the biotin-labeled DNA oligonucleotide with complementary sequence to the target small RNA **(A)**. The mixture of total small RNA and biotin-labeled DNA oligonucleotide was then incubated with streptavidin magnetic particle **(B)** followed by magnetic separation **(C)**. In step 2, the purified small RNA from Step 1 was treated with exonuclease I to degrade the possible residual DNA oligonucleotide **(D)**, and next incubated with a DNA antisense to form RNA/DNA duplex **(E)**. The sample was then incubated with nuclease S1 to eliminate the incomplete complementary small RNAs **(F)**. Finally, the purified small RNA from step f was treated with Dnase I to eliminate the DNA antisense **(G)**.

The main obstacle of detection of small RNA modification is to purify specific small RNA to achieve the purity required by LC-MS/MS. Currently, the only major method to purify specific small RNA is the pulldown assay based on magnetic beads with single-stranded DNA/RNA (ssDNA/RNA) capture probes (Berensmeier, 2006; Adams et al., 2015; Zhou et al., 2021). However, due to the great sequence similarity among different small RNAs, the efficiency and purity of isolated target miRNA *via* RNA pulldown assay are often not qualified for LC-MS/MS analysis. In order to detect small RNA modification, especially 2′-O-methylation, we developed a two-step strategy (RNA pulldown assay-step 1 and enzyme protection assay-step 2) to purify the target small RNA from tissue or cell samples for LC-MS/MS analysis (Figure 1). In step 1, we carried out the small RNA purification with the biotin-labeled DNA oligonucleotide. This DNA oligonucleotide is antisense to the target small RNA but also contains two extra A residues at its 5′-end. Total small RNA was incubated with the biotinylated DNA in 0.5× SSC at 50°C for 15 h. Then, the biotinylated DNA was captured by streptavidin magnetic particle (Roche) and washed with ×0.5 SSC. The target small RNA was then eluted by DEPC water (5 min, 70°C). In step 2, the purified target small RNA was treated with exonuclease I (Amersham Biosciences) for 1 h to degrade the DNA oligonucleotide that might be eluted. The quality and amount of small RNA were evaluated by RT-qPCR according to the synthetic RNA standard. The 21-nt single-stranded DNA with sequence complementary to the target small RNA (2µL, 10 µM) was added in buffer (20 nM Tris–HCl, 100 mM KCl, 5mM MgCl_2_, pH 7.5) containing 10 µg target small RNA. The mixture was incubated at 95°C for 2 min followed by decreasing to 25°C slowly. To eliminate unpaired RNAs, the mixture was treated with 1000 units nuclease S1 (Thermo Fisher) (30 min, room temperature and terminated by incubation at 70°C for 10 min). Finally, the 21-nt single-stand DNA was then degraded by treatment with 4 units DNaseI (New England Biolabs) at 37°C for 30 min. The remaining small RNA was then extracted by RNA Clean and Concentrator-5 kit (Zymo Research) according to the manufacturer’s instructions. The quality of the purified target small RNA was evaluated by qRT-PCR or LC-MS/MS.

To validate this strategy, we synthesized non-methylated miR-21-2p, miR-26-5p, piR-020485, and tsRNA, as well as methylated miR-21-2p^CH3^, miR-26-5p^CH3^, piR-020485^CH3^, and tsRNA^CH3^ at the 3′-end to serve as the standards. Supplementary Figure S1 shows the standard curve of stem-loop probe RT-qPCR for miR-21-5p/miR-21-5p^CH3^, miR-26-5p/miR-26-5p^CH3^, piR-020485/piR-020485^CH3^, and tsRNA/tsRNA^CH3^. These synthetic small RNAs were analyzed by LC-MS/MS. As shown in Supplementary Figure S2, synthetic methylated miR-21-5p^CH3^, miR-26-5p^CH3^, piR-020485^CH3^, and tsRNA^CH3^ exhibited a peak “shift” of a methyl group compared to non-methylated miR-21-5p, miR-26-5p, piR-020485, and tsRNA, respectively.

**FIGURE 2:**
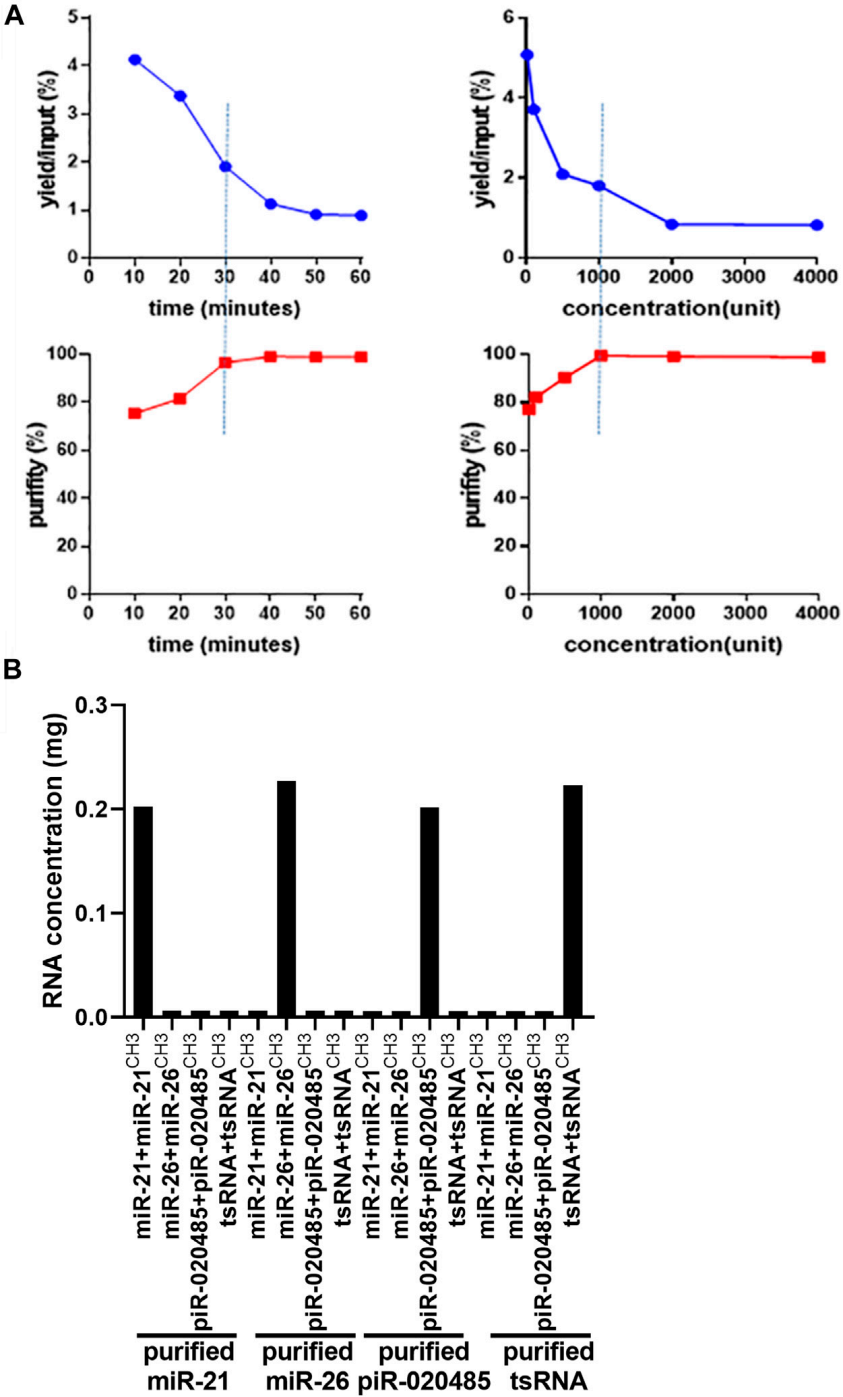
Optimized condition of nuclease S1 treatment in the procedure of small RNA purification. **(A)** The yield and purity of small RNAs isolated from the mixture of synthetic small RNAs by treating small RNAs with nuclease S1 treatment for different times (left) or at various concentrations (right). **(B)** Stem–loop probe-based RT-qPCR analysis of concentration of purified miR-21-5p/miR-21-5p^CH3^, miR-26-5p/miR-26-5p^CH3^, piR-020485/piR-020485^CH3^, and tsRNA/tsRNA^CH3^ after treatment with 1000 units nuclease S1 for 30 min Unit refers to the amount of enzyme that catalyzes the conversion of 1 micromole (μmole) of substrate per minute.

Next, we mixed these synthetic small RNAs equally and employed our two-step method to purify individual small RNA from the mixture. As treatment with nuclease S1 is critical in controlling the purity and yield of target small RNA, we optimized the condition of nuclease S1 treatment *via* monitoring the purity and yield of isolated target small RNA under the different incubation times and concentrations of nuclease S1. As shown in Figure 2A, the yield and purity of isolated target small RNAs were time- and dose-dependent upon nuclease S1 treatment**.** Considering the purity and yield of isolated small RNA, we optimized the treatment condition of nuclease S1 as 1000 units enzyme and 30 min incubation**.** Under this condition, miR-21-5p/miR-21-5p^CH3^, miR-26-5p/miR-26-5p^CH3^, piR-020485/piR-020485^CH3^, or tsRNA/tsRNA^CH3^ were successfully purified from the mixture of synthetic small RNAs, as shown by stem-loop RT-qPCR analysis (Figure 2B).

**FIGURE 3:**
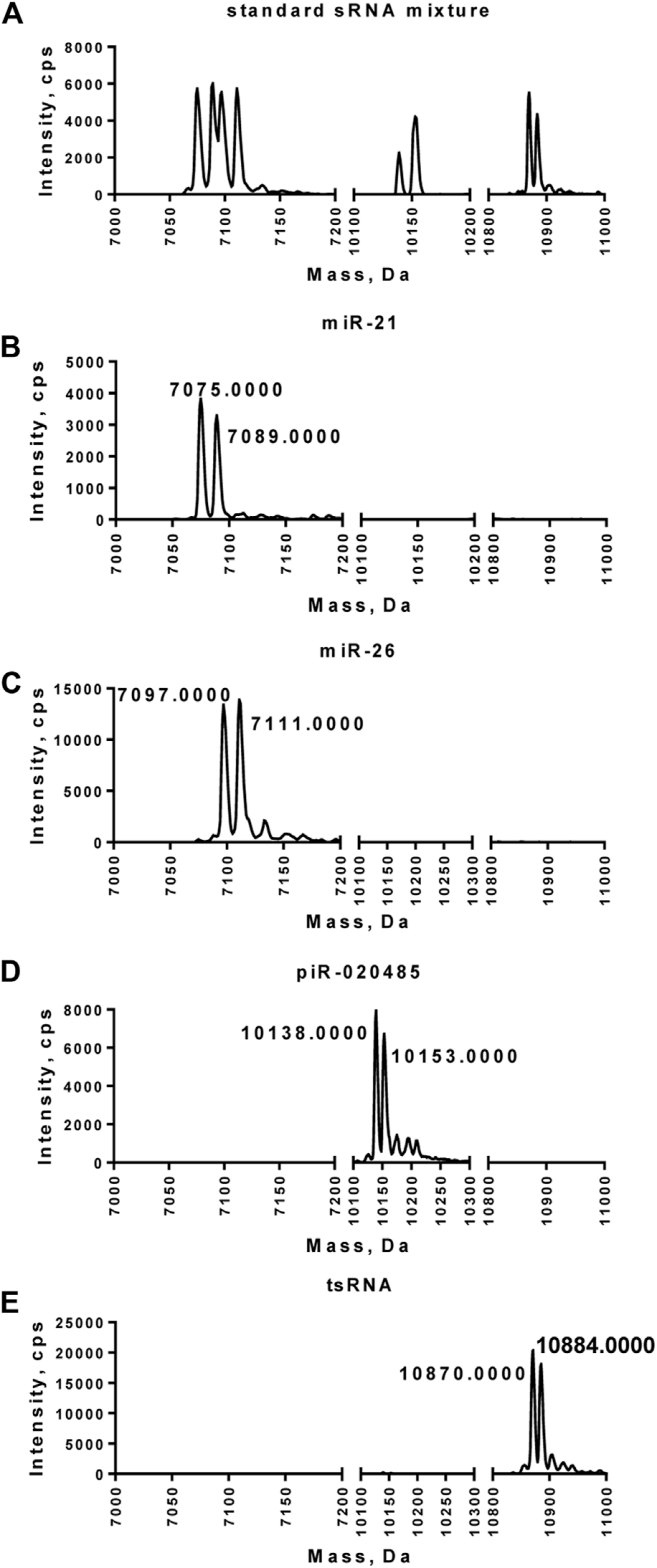
Mass spectrometry analysis of miR-21-5p/miR-21-5p^CH3^, miR-26-5p/miR-26-5p^CH3^, piR-020485/piR-020485^CH3^, and tsRNA/tsRNA^CH3^ purified from the mixture of synthetic small RNAs. **(A)** Synthetic methylated or unmethylated small RNA mixture detected by LC-MS/MS. **(B–E)** Purified miR-21-5p/miR-21-5p^CH3^ **(B)**, miR-26-5p/miR-26-5p^CH3^ **(C)**, piR-020485/piR-020485^CH3^ **(D)**, and tsRNA/tsRNA^CH3^ **(E)** detected by LC-MS/MS. The position of a peak along the *x*-axis represented the molecular mass of the species. Signal intensity on the *y*-axis represented the amount of small RNA in each peak.

LC-MS/MS analysis was performed next to characterize the paired miR-21-5p/miR-21-5p^CH3^, miR-26-5p/miR-26-5p^CH3^, piR-020485/piR-020485^CH3^, or tsRNA/tsRNA^CH^ purified from the mixture of synthetic small RNAs. Compared to the small RNA mixture, which exhibits multiple peaks ranging from 7050 to 10900 Daltons, purified miR-21-5p/miR-21-5p^CH3^, miR-26-5p/miR-26-5p^CH3^, piR-020485/piR-020485^CH3^, or tsRNA/tsRNA^CH^ displayed two peaks at 7075–7089, 7097–7111, 10,138–10153, or 10,870–10884 Daltons, respectively (Figures 3A–E). Among these two peaks, the peak with smaller mass represented the non-methylated small RNAs, while the peak with larger mass represented the methylated small RNAs.

### Isolation of small RNAs from human lung tissue

**FIGURE 4:**
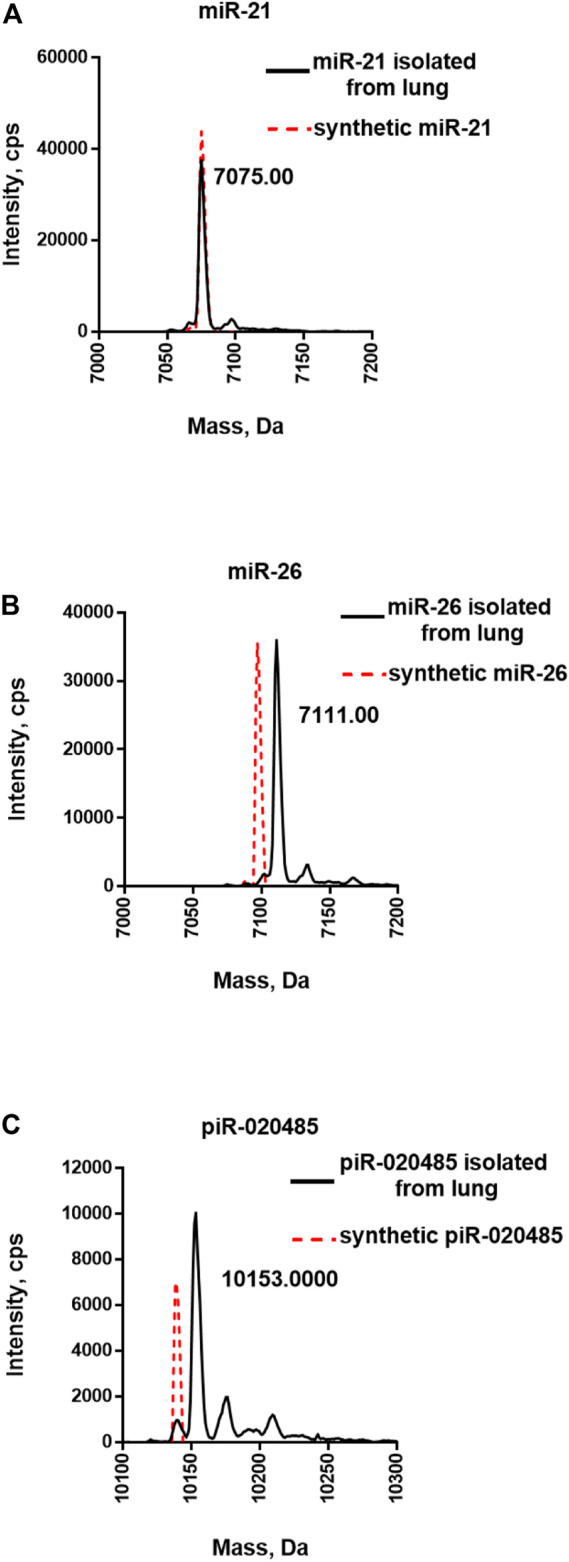
Mass spectrometry analysis of miRNAs and piRNA directly purified from human normal lung tissues. **(A)** LC-MS/MS analysis of purified miR-21-5p. **(B)** LC-MS/MS analysis of purified miR-26-5p. **(C)** LC-MS/MS analysis of purified piR-020485. The synthetic unmethylated miR-21-5p, miR-26-5p, and piR-020485 (red dot lines) served as the controls.

Our previous study confirmed that miRNAs in human lung tissue are selectively methylated at the 3′-end (Liang et al., 2020a). Next, we extracted small RNAs from the human normal lung tissue and purified miR-21-5p, miR-26-5p, or piR-020485 using the two-step strategy. To validate the purity of the isolated target miR-21-5p, miR-26-5p, and piR-020485, three different rRNAs (5, 5.8, and 28S rRNAs) were probed by qRT-PCR since these rRNAs comprise a majority of the cellular RNA and represent the most likely nonspecific contaminant in purification due to their abundance. We found that 99.99% of those rRNAs were removed (Supplementary Figure S3), illustrating the specificity of our two-step strategy. LC-MS/MS analysis was performed to characterize the purified small RNAs. Purified miR-21-5p displayed one major peak with a mass of 7075 Daltons, the same mass of synthetic non-methylated miR-21-5p (Figure 4A and Supplementary Figure S4A). In contrast, a peak shift from the mass of 7075 Da (synthetic non-methylated, red dot line) to 7111 Da was detected in purified miR-26-5p, indicating the purified miR-26-5p from human normal lung tissue is methylated (Figure 4B and Supplementary Figure S4B). These results agree with our recent report that miR-21-5p in human normal lung tissues is non-methylated, while miR-26-5p is methylated at the 3′-end (Liang et al., 2020a). As mature piRNAs in animals and humans also possess the 3′-terminal 2′Ome (Ohara et al., 2007), we next purified piR-020485 from human normal sperm samples using the two-step strategy. As shown in Figure 4C, the purified piR-020485 displayed a peak with a mass of 10,153 Daltons, larger than synthetic non-methylated piR-020485 (red dot line), confirming the 3′-terminal 2′Ome of purified piR-020485 from human sperm (Figure 4C and Supplementary Figure S4C).

### Isolation of tsRNA from human sperm

**FIGURE 5:**
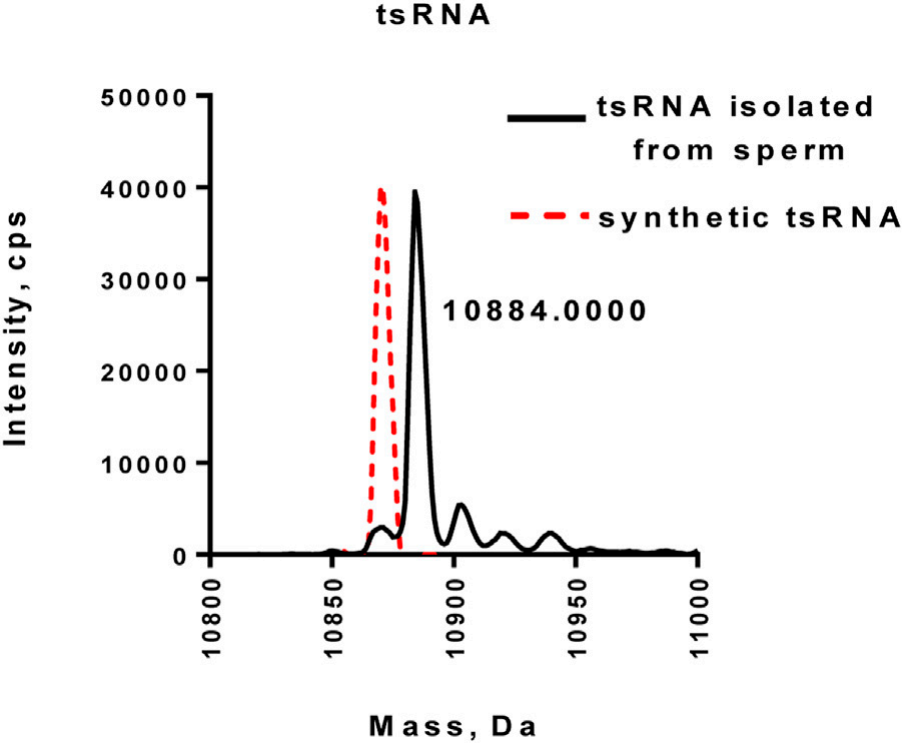
Mass spectrometry analysis of tsRNA directly purified from human normal sperm. The synthetic unmethylated tsRNA (red dot lines) served as the control.

Recent studies showed that several sperm tRNA-derived small RNAs (tsRNAs) were involved in the intergenerational transmission of disorders (Chen et al., 2016; Sharma et al., 2016). The study by Schaefer et al. demonstrated that tRNAs, including tRNA^Asp(GTC)^, tRNA^Val(AAC)^, and tRNA^Gly(GCC)^, were able to be methylated by Dnmt2, which, in turn, protected tRNAs from cleavage under stress conditions (Schaefer et al., 2010). To test whether tsRNAs in human sperms (tRNA^His(GTG)^) are methylated at the 3′-end, we purified tsRNA from human sperms by the two-step strategy and performed LC-MS/MS analysis of purified tsRNA. As shown in Figure 5, the purified tsRNA exhibited a peak with a mass of 10,884 Daltons, which was larger than synthetic non-methylated tsRNA (red dot line), confirming that tsRNAs purified from human sperm possessed 2′Ome modification at the 3′-end (Figure 5 and Supplementary Figure S4D).

## Discussion

Previous studies by us and others have confirmed that mammalian small RNAs, such as piRNA and miRNA, are 2′-O-methylated at the 3′-end nucleotide (Yu et al., 2005; Ohara et al., 2007; Liang et al., 2020a). Given that small RNAs play vital roles in various biological processes in humans and animals and that the 3′terminal 2′Ome of small RNAs is critical for the biogenesis and function of small RNAs, it is necessary to determine the 2′-O-methylation of small RNAs accurately. In this study, we developed a two-step strategy, combining RNA pulldown (step 1) and enzyme protection assay (step 2) to purify small RNAs directly from human tissue samples. Using this method, we have successfully purified miR-21-5p, miR-26-5p, piR-020485, and tsRNA from human lung and sperm tissue samples, respectively. By LC-MS/MS analysis, we further characterized the 2′-O-methylation at the 3′-end nucleotide of the purified target small RNAs.

The current method for identifying the 2′-O-methylation of small RNA is sodium periodate oxidation in combination with Northern blot or RT-qPCR (Yu et al., 2005; Ohara et al., 2007; Wang et al., 2018). However, all these methods are indirect proof of small RNA methylation and cannot identify the accurate position of methylation. Therefore, many attempts have been made to develop new methods for analyzing small RNA methylation. The big obstacle preventing the success of these methods is the purification of the target small RNA. Most previous studies performed RNA immunoprecipitation using oligonucleotide, which is complementary to target small RNA (Yu et al., 2005; Ohara et al., 2007; Liang et al., 2020a). However, unlike large RNA fragments, small RNAs generally share high similarity in sequence. For instance, different miRNAs may have 20 identical nucleotides out of 21 nucleotides (Kozomara et al., 2019). The purity of isolated target miRNA by RNA immunoprecipitation method often failed to achieve the degree by LC-MS/MS analysis. To overcome this problem, we developed a two-step strategy for purifying specific small RNA from human tissue samples by combining RNA immunoprecipitation and enzyme protection assay. In step 1, RNA immunoprecipitation was performed to isolate target small RNA from the total small RNAs extracted from tissues. According to our results and previous report (Yu et al., 2005), the purity of isolated target small RNA by such pulldown assay could achieve ∼70%. To increase the purity of isolated target small RNA, in step 2, we used a single-stranded DNA, with a sequence completely complementary to the target small RNA, to mix with the isolated target small RNA and form a DNA/RNA hybrid double-strand, which would protect the target small RNA in the following sequential digestion procedure. The unpaired small RNAs were digested by nuclease S1, and the extra single-stranded DNA and the DNA strand in the DNA/RNA hybrid duplex were cleaved by Dnase I. After a sequential digestion procedure, the purity of the isolated target small RNA could reach 99% and was ready for LC-MS/MS analysis. In summary, we established a novel two-step method to purify specific small RNAs from human tissues for methylation analysis by LC-MS/MS.

## Data Availability

The raw data supporting the conclusion of this article will be made available by the authors without undue reservation.

## Abbreviations

DEPC, diethyl pyrocarbonate; CH3, methyl group; LC-MS/MS: liquid chromatography-coupled with mass spectrometry; 2′Ome, 2′-O-methylation; OH, hydroxyl; 2D-TLC, two-dimensional thin-layer chromatography; SSC, saline sodium citrate buffer.
