# Supplementary material for: A novel method to purify small RNAs from human tissues for methylation analysis by LC-MS/MS
Source: Front Mol Biosci. 2022 Aug 30;9:949181. doi: 10.3389/fmolb.2022.949181 (PMC9468635; doi:10.3389/fmolb.2022.949181)
Supplement: Supplementary file 1 [file DataSheet1.docx]

Supplementary materials for

**A novel method to purify small RNAs from human tissues for methylation analysis by LC-MS/MS**

Rong Yang^1*^, Jianfeng Li^1*^, Yifan Wu^1^, Xinli Jiang^1^, Shuang Qu^2^, Qiang Wang^1¶^, Hongwei Liang^2¶^, and Ke Zen^1¶^

**File list**

1. Supplementary Table 1
2. Supplementary Figure 1
3. Supplementary Figure 2
4. Supplementary Figure 3

**Supplementary Table 1. The sequences of oligonucleotides used in the experiment.**

| **sRNA** | **Sequence (5'-3')** |
| --- | --- |
| miR-21-5p | UAGCUUAUCAGACUGAUGUUGA |
| miR-21-5p^CH3^ | UAGCUUAUCAGACUGAUGUUGAm |
| miR-26-5p | UUCAAGUAAUCCAGGAUAGGCU |
| miR-26-5p^CH3^ | UUCAAGUAAUCCAGGAUAGGCUm |
| piR-020485 | TGGTGCAGGACAGTGTGTCTCCTGGAC |
| piR-020485^CH3^ | TGGTGCAGGACAGTGTGTCTCCTGGACm |
| tsRNA | GATTGTGAATCTGACAACAGAGGCTTACGACCCC |
| tsRNA^CH3^ | GATTGTGAATCTGACAACAGAGGCTTACGACCCCm |
| 28S forward primer | CAGGGGAATCCGACTGTTTA |
| 28S reverse primer | ATGACGAGGCATTTGGCTAC |
| 5S forward primer | GTCTACGGCCATACCACCCTG |
| 5S reverse primer | GCCTACAGCACCCGGTATTCC |
| 5.8S forward primer | ACTCGGCTCGTGCGTC |
| 5.8S reverse primer | GCGACGCTCAGACAGG |

**Supplementary Figure 1.** **The standard curve of Stem–loop probe-based RT-qPCR assay for synthetic miR-21-5p/miR-21-5p^CH3^,** **miR-26-5p/miR-26-5p^CH3^,** **piR-020485/piR-020485^CH3^ and tsRNA/tsRNA^CH3^.** RNA concentration represents the concentration of synthetic RNAs before reverse transcription.

**Supplementary Figure 2. Detection of synthetic miR-21-5p/miR-21-5p^CH3^, miR-26-5p/miR-26-5p^CH3^, piR-020485/piR-020485^CH3^ and tsRNA/tsRNA^CH3^ by Mass spectrometry.**

**
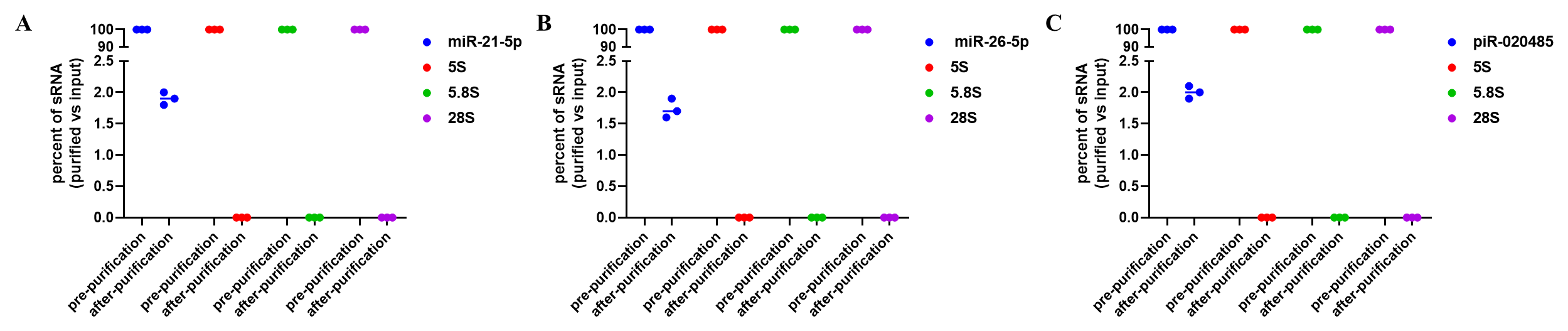
**

**Supplementary Figure 3. Detection of target small RNA and 5S, 5.8S, and 28S rRNAs before and after purification from lung tissues.** (A) The percentage of miR-21-5p, 5S, 5.8S, and 28S rRNAs before and after miR-21-5p purification from lung tissues. (B) The percentage of miR-26-5p, 5S, 5.8S, and 28S rRNAs before and after miR-26-5p purification from lung tissues. (C) The percentage of piR-020485, 5S, 5.8S, and 28S rRNAs before and after piR-020485 purification from lung tissues.

**
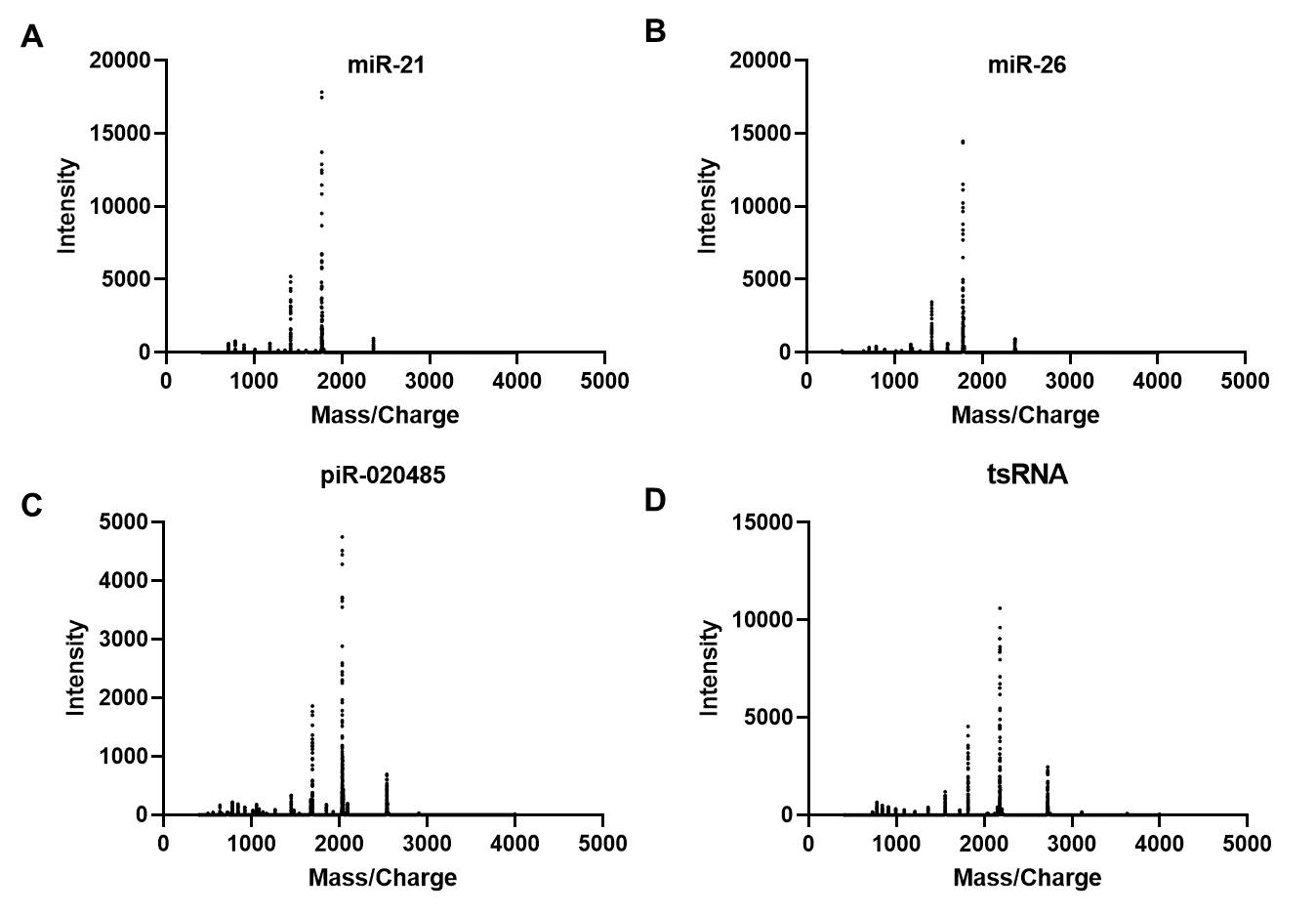
 Supplementary Figure 4. LC-MS/MS of small RNAs isolated from lung or sperm. A-C) MS of** miR-21-5p, miR-26-5p and piR-020485 isolated from lung. **D) MS of** tsRNA isolated from sperm.
